# Characterization of Marangoni Forced Convection in Casson Nanoliquid Flow with Joule Heating and Irreversibility

**DOI:** 10.3390/e22040433

**Published:** 2020-04-10

**Authors:** Muhammad Adil Sadiq, Tasawar Hayat

**Affiliations:** 1 Department of Mathematics, DCC-KFUPM Box 5084, Dhahran 31261, Saudi Arabia; 2 Department of Mathematics, Quaid-I-Azam University, Islamabad 45320, Pakistan; fmgpak@gmail.com; 3 Nonlinear Analysis and Applied Mathematics (NAAM) Research Group, Department of Mathematics, Faculty of Science, King Abdulaziz University, Jeddah 21589, Saudi Arabia

**Keywords:** mixed convection, rotating cone, viscous fluid, Bejan number, entropy generation, thermal radiation, viscous dissipation, Dufour and Soret effects and chemical reaction

## Abstract

The Marangoni forced convective inclined magnetohydrodynamic flow is examined. Marangoni forced convection depends on the differences in surface pressure computed by magnetic field, temperature, and concentration gradient. Casson nanoliquid flow by an infinite disk is considered. Viscous dissipation, heat flux, and Joule heating are addressed in energy expressions. Thermophoresis and Brownian motion are also examined. Entropy generation is computed. The physical characteristics of entropy optimization with Arrhenius activation energy are discussed. Nonlinear PDE’s are reduced to highly nonlinear ordinary systems with appropriate transformations. A nonlinear system is numerically computed by the NDSolve technique. The salient characteristics of velocity, temperature, concentration, entropy generation, and Bejan number are explained. The computational results of the heat-transfer rate and concentration gradient are examined through tables. Velocity and temperature have reverse effects for the higher approximation of the Marangoni number. Velocity is a decreasing function of the Casson fluid parameter. Temperature is enhanced for higher radiation during reverse hold for concentration against the Marangoni number. The Bejan number and entropy generation have similar effects for Casson fluid and radiation parameters. For a higher estimation of the Brinkman number, the entropy optimization is augmented.

## 1. Introduction

An investigation of Marangoni forced convection is of great interest for the dissipative boundary layer between a two-phase liquid flow like liquid–liquid and gas–liquid interfaces. Marangoni convection depends on the difference in surface pressure determined by the concentration gradient, magnetic field, and temperature gradient. These gradients occur when the liquid boundary layer has different characteristics. Few significant applications of the Marangoni forced convection impact include thin-film stretching, material sciences, applied physics, silicon wafers, nanotechnology, semiconductor processing, soap films, etc. In addition, melting and welding are much more efficient manufacturing applications of the Marangoni convection concept. Heat transfer in the Marangoni boundary layer flow is also comprehensively deliberated. For instance, the magnetohydrodynamic Marangoni convection flow of Casson axisymmetric nanomaterials with Joule heating is presented by Shafiq et al. [1]. Rasool et al. [2] examined magnetohydrodynamic (MHD) Marangoni forced convection flow of second-grade nanomaterials with Brownian diffusion and thermophoresis. Heat flux in Marangoni convective flow of a carbon nanotube was examined by Hayat et al. [3]. Imai et al. [4] developed a numerical analysis of Marangoni convective nanoscale flow. Prominent behavior of the thermal transfer rate in the Marangoni boundary flow of pseudoplastic nanoliquids filling a porous medium is given by Lin et al. [5]. Some relevant studies about Marangoni convective flow are highlighted in refs. [6,7,8].

It is noticed that flow in the presence of a chemical reaction is discussed extensively. However, few studies present in this direction when activation energy becomes significant. In fact, the magnitude of the energy barrier separates the minimum potential energy surface concerning the initial and final thermodynamic state. It can be affected by catalysts and temperature. Activation energy fluctuates for different chemical reactions. Activation energy with a chemical reaction has applications in the fields of water emulsions and geothermal engineering. Bestman [9] introduced the concept of activation energy in boundary layer flows. He presented a study for Arrhenius activation energy on mass transfer. The effects of entropy optimization and activation energy in flow are studied by Hayat et al. [10]. Hamid et al. [11] explored the activation energy in unsteady flow of Williamson nanomaterial as illustrated. Khan et al. [12] studied the activation energy for the stagnation point flow of Cross nanofluids. Characteristics of magnetohydrodynamic Couette–Poiseuille nanomaterial flows with activation energy are deliberated by Zeeshan et al. [13]. Khan et al. [14] discussed activation energy in Prandtl–Eyring nanomaterial with chemical reactions. Kumar et al. [15] investigated the influence of activation energy in magnetohydrodynamic flows of Carreau liquids over a stretching surface. Activation energy and the entropy impact in tangent hyperbolic nanoliquid flows are presented by Khan et al. [16]. Thermophoresis and Brownian diffusion in convective nanomaterial flow with activation energy are addressed by Dhlamini et al. [17]. Some advancements about heat and mass transfer are highlighted in refs. [18,19,20,21,22,23,24].

Recently, one of the most important concerns for engineers and scientists was to develop a mechanism that controls the consumption of beneficial energy. All thermal devices depend on the thermodynamic principle and create an irreversibility phenomenon. The use of the second thermodynamic law leads to a mathematical technique for the reduction of entropy-generation rate and friction. It is one of the methods that can be used to determine the destruction in the current performance of a thermal system. Therefore, it is essential to improve entropy optimization to inhibit any thermal losses that can disturb system performance. The idea of entropy-generation minimization is necessary to improve efficiency in thermodynamic systems such as thermal storage, the design of heat exchangers, power plants, the cooling of electronic devices, the environmental control of aircrafts, refrigerators, etc. Entropy optimization has attained more consideration due to tremendous applications in power collectors, geothermal energy systems, engineering phenomena, geothermal processes, slider bearings, fuel cells, and advanced nanotechnology. Therefore, in recent times, numerous engineers and scientists have focused their attention on entropy-generation complications. This has enhanced the significance of many electronic devices and led to engineering improvements. Bejan [25,26,27,28] deliberated through theoretical works about entropy in fluid flow with thermal transport. He revealed a new concept for the significance of the thermal system, which acts as the thermodynamic principle. Gibanov et al. [29] explained MHD natural convective flow of nanoliquid with entropy optimization. Irreversibility exploration in Marangoni convective flow of Newtonian liquid in an open cavity is investigated by Saleem et al. [30]. Viscous entropy for carbon nanotube-based Darcy–Forchheimer nanoliquid flow is exemplified by Khan et al. [31]. An investigation of entropy in non-Newtonian nanomaterial flow is explored by Zhuang and Zhu [32]. Numerical analysis of irreversibility in a rectangular convective porous fin is discussed by Khatami and Rahbar [33]. Khan et al. [34] described the entropy rate in MHD nanoliquid flow between two stretchable rotating disks.

The above-mentioned literature survey witnesses that no attempt has been made to deliberate the influence of entropy generation in Marangoni forced convection Casson nanoliquid flow by an infinite disk. Here we address such a problem. The thermal and solutal capillaries’ behaviors are key factors in Marangoni convection of liquids and nanoliquids. Thermophoresis and Brownian diffusion behaviors are scrutinized. Entropy generation and Arrhenius activation energy are accounted for. A nonlinear ordinary differential system is computed by the NDSolve technique. Characteristics of different involved parameters for velocity, temperature, concentration, entropy optimization, and Bejan number are examined. The computational outcome of Nusselt and Sherwood number are numerically deliberated. Reasonable agreement is found between the present and previous results in a limiting sense (see Table 1).

## 2. Statement of Problem

**Figure 1:**
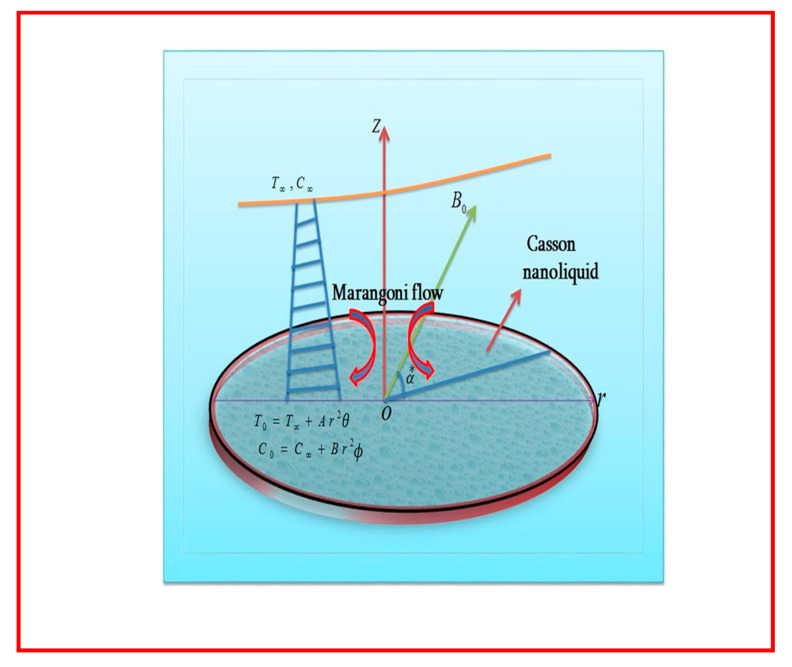
Flow diagram.

Marangoni forced convective inclined magnetohydrodynamic flow of Casson nanoliquid by an infinite disk is studied. Flow generated due to concentration and temperature gradients is also studied. Thermal radiation, Joule heating, and viscous dissipation in energy equation are considered. The thermal and solutal capillaries’ behaviors are key components in Marangoni convection of liquid and nanoliquid. Impacts of thermophoresis and Brownian diffusion are discussed. Salient features of entropy optimization and Arrhenius activation energy are explained. The current density (J) is Ohm’s laws is J = σ(E + V × B). Here, E represents the strength of the electric field, which is neglected; B is magnetic field strength, and σ is electrical conductivity. Induced magnetic field and Hall effects for low-magnetic Reynolds numbers are neglected. The ionslip effects are ignored. The magnetic field of constant strength ($B_{0}$) is exerted. The linearized form of thermal radiation is taken. The geometry of the problem is presented in Figure 1.

Employing continuity, momentum, energy, and concentration expressions [2,3,6]:

(1) $$\frac{\partial u}{\partial r}+\frac{u}{r}+\frac{\partial w}{\partial z}=0,$$

(2) $$u\frac{\partial u}{\partial r}+w\frac{\partial u}{\partial z}=\frac{\mu }{\rho }\left(1+\frac{1}{\beta }\right)\frac{\partial^{2}u}{\partial z^{2}}-\frac{\sigma^{⊗}B_{0}^{2}}{\rho }\sin\alpha^{*}u,$$

(3) $$\begin{array}{c} u\frac{\partial T}{\partial r}+w\frac{\partial T}{\partial z}=\frac{k}{\left(\rho c_{p}\right)}\frac{\partial^{2}T}{\partial z^{2}}+\frac{\mu }{\left(\rho c_{p}\right)}\left(1+\frac{1}{\beta }\right){\left(\frac{\partial u}{\partial z}\right)}^{2}+\tau \frac{D_{T}}{T_{\infty }}{\left(\frac{\partial T}{\partial z}\right)}^{2}+\tau D_{B}\frac{\partial T}{\partial z}\frac{\partial C}{\partial z}\\ +\frac{16}{3}\frac{\sigma^{*}T_{\infty }^{3}}{k^{*}\left(\rho c_{p}\right)}\frac{\partial^{2}T}{\partial z^{2}}+\frac{\sigma^{⊗}B_{0}^{2}}{\left(\rho c_{p}\right)}\sin\alpha^{*}u^{2}, \end{array}\}$$

(4) $$u\frac{\partial C}{\partial r}+w\frac{\partial C}{\partial z}=D_{B}\frac{\partial^{2}C}{\partial z^{2}}+\frac{D_{T}}{T_{\infty }}\frac{\partial^{2}T}{\partial z^{2}}-k_{r}^{2}{\left(\frac{T}{T_{\infty }}\right)}^{n}\exp[\frac{-E_{a}}{KT}]\left(C-C_{\infty }\right)$$

The boundary conditions are

(5) $$\begin{array}{c} \mu \left(1+\frac{1}{\beta }\right)\frac{\partial u}{\partial z}=\frac{\partial \sigma }{\partial T}\frac{\partial T}{\partial r}+\frac{\partial \sigma }{\partial C}\frac{\partial C}{\partial r},\;w=0,\\ T_{w}=T_{0}r^{2}=T_{\infty }+Ar^{2}\theta ,\;C_{w}=C_{0}r^{2}=C_{\infty }+Br^{2}\phi ,\;\mathrm{at}\;z=0\\ u\to 0,\;T=T_{\infty },\;C=C_{\infty }\;\mathrm{when}\;z\to \infty \end{array}\}$$

where μ indicates the dynamic viscosity, u and w are the velocity components, ρ is density, r and z are cylindrical coordinates, $T_{w}\;\mathrm{and}\;C_{w}$ are temperature and concentration of disk, β is Casson fluid parameter, $\sigma^{⊗}$ is electrical conductivity, $\alpha^{*}$ is inclination angle, σ is surface tension, k is thermal conductivity, T is temperature, $c_{p}$ is specific heat, $T_{\infty }$ is ambient temperature, τ is ratio of heat capacities, $k^{*}$ is mean absorption coefficient, $D_{T}$ is thermophoresis diffusion coefficient, $\sigma^{*}$ is Stefan–Boltzman constant, $D_{B}$ is Brownian movement coefficient, C is concentration, $k_{r}$ is reaction rate, $C_{\infty }$ is ambient concentration, $E_{a}$ is activation energy, n is fitted rate constant, $K=8.61\times 10^{-5}eV/K$ is Boltzman constant, ${\left(\frac{T}{T_{\infty }}\right)}^{n}\exp[\frac{-E_{a}}{KT}]$ is Arrhenius function, and a is positive dimensional constant.

We consider surface tension as a linear function of concentration and temperature [1,2]:

(6) $$\sigma =\sigma_{0}-\gamma_{T}\left(T-T_{\infty }\right)-\gamma_{C}\left(C-C_{\infty }\right),$$

with

(7) $$\gamma_{T}={-\frac{\partial \sigma }{\partial T}|}_{T=T_{\infty }},\;\gamma_{C}={-\frac{\partial \sigma }{\partial C}|}_{C=C_{\infty }}.$$

where $\gamma_{T}$, $\sigma_{0}$, and $\gamma_{C}$ denote the positive constants.

Taking

(8) $$\begin{array}{c} u=r\Omega f^{\prime }\left(\xi \right),\;w=-2\sqrt{\Omega \nu }f\left(\xi \right),\;\theta \left(\xi \right)=\frac{\left(T-T_{\infty }\right)}{T_{0}r^{2}}\\ \phi \left(\xi \right)=\frac{\left(C-C_{\infty }\right)}{C_{0}r^{2}},\;\xi =\sqrt{\frac{\Omega }{\nu }}z \end{array}\},$$

we obtain

(9) $$\left(1+\frac{1}{\beta }\right)f^{\prime \prime \prime }+2ff^{\prime \prime }-f^{\prime 2}-M\sin^{2}\alpha^{*}f^{\prime }=0,$$

(10) $$\left(1+Rd\right)\theta^{\prime \prime }-2\mathrm{Pr}f^{\prime }\theta +2\mathrm{Pr}f\theta^{\prime }+\mathrm{Pr}Nt{\theta^{\prime }}^{2}+\mathrm{Pr}Nb\theta^{\prime }\phi^{\prime }+Br\left(1+\frac{1}{\beta }\right){f^{\prime \prime }}^{2}+M\sin^{2}\alpha^{*}{f^{\prime }}^{2},$$

(11) $$\phi^{\prime \prime }-2f^{\prime }\phi +2Lef\phi^{\prime }+\frac{Nt}{Nb}\theta^{\prime \prime }-\lambda Le\phi \left(1+n\alpha_{1}\theta \right)\exp[\frac{-E}{\left(1+\alpha_{1}\theta \right)}],$$

(12) $$\begin{array}{c} f\left(0\right)=0,\;\left(1+\frac{1}{\beta }\right)\;f^{\prime \prime }\left(0\right)=-2M_{a}\left(1+R_{a}\right),\;\theta \left(0\right)=1,\;\phi \left(0\right)=1\\ f^{\prime }\left(\infty \right)=0,\;\theta \left(\infty \right)=0,\;\phi \left(\infty \right)=0 \end{array}\}$$

Here, $M\left(=\frac{\sigma^{⊗}B_{0}^{2}}{\Omega \rho }\right)$ represents magnetic parameter, $M_{a}\left(=\frac{\gamma_{T}A}{\mu \Omega }\sqrt{\frac{\Omega }{\gamma }}\right)$ Marangoni number, $Rd\left(=\frac{16}{3}\frac{\sigma^{*}T_{\infty }^{3}}{k^{*}k}\right)$ radiation parameter, $R_{a}\left(=\frac{\gamma_{C}B}{\gamma_{T}A}\right)$ Marangoni ratio parameter, $\mathrm{Pr}\left(=\frac{\nu }{\alpha }\right)$ Prandtl number, $Ec\left(=\frac{\Omega^{2}}{c_{p}T_{0}}\right)$ Eckert number, $Nb\left(=\frac{\tau D_{B}C_{0}}{\nu }\right)$ Brownian diffusion parameter, Br(=PrEc) Brinkman number, $Nt\left(=\frac{\tau D_{T}T_{0}}{\nu T_{\infty }}\right)$ thermophoresis parameter, $Le\left(=\frac{v}{D}\right)$ Lewis number, $\lambda \left(=\frac{k_{r}^{2}}{\Omega }\right)$ chemical reaction parameter, $E\left(=\frac{E_{a}}{KT_{\infty }}\right)$ activation energy parameter, and $\alpha_{1}\left(=\frac{T_{0}}{T_{\infty }}\right)$ temperature difference variables.

## 3. Entropy Modeling

Entropy generation is defined as [30,31,34]:

(13) $$\begin{array}{c} S_{G}=\frac{k}{T_{\infty }^{2}}\left(1+\frac{16}{3}\frac{\sigma^{*}T_{\infty }^{3}}{k^{*}k}\right){\left(\frac{\partial T}{\partial z}\right)}^{2}+\frac{\mu }{T_{\infty }}\left(1+\frac{1}{\beta }\right){\left(\frac{\partial u}{\partial z}\right)}^{2}+\frac{\sigma^{⊗}B_{0}^{2}}{T_{\infty }}\sin^{2}\alpha^{*}u^{2}\\ +\frac{RD}{T_{\infty }}\left(\frac{\partial T}{\partial z}\frac{\partial C}{\partial z}\right)+\frac{RD}{C_{\infty }}{\left(\frac{\partial C}{\partial z}\right)}^{2} \end{array}\},$$

it should be noted that in the above expression, the entropy generation is because of four effects. The first term on RHS is due to the heat transfer across a finite temperature difference. It is a local volumetric entropy generation known as heat transfer irreversibility (HTI). The second term, known as fluid friction irreversibility, occurs because of viscous dissipation. The third term occurs in view of the magnetic field. The last two terms indicate diffusion irreversibility. These are because of diffusion or mass transfers across finite concentration differences.

In dimensionless variables we have

(14) $$N_{G}=\alpha_{1}\left(1+Rd\right){\theta^{\prime }}^{2}+Br\left(1+\frac{1}{\beta }\right){f^{\prime \prime }}^{2}+MBr\sin^{2}\alpha^{*}{f^{\prime }}^{2}+L\theta^{\prime }\phi^{\prime }+L\frac{\alpha_{2}}{\alpha_{1}}{\phi^{\prime }}^{2}.$$

## 4. Bejan Number

The Bejan number is defined as

(15) $$Be=\frac{\mathrm{Heat}\;\mathrm{and}\;\mathrm{mass}\;\mathrm{transfer}\;\mathrm{irreversibility}}{\mathrm{Total}\;\mathrm{irreversibility}},$$

or

(16) $$Be=\frac{\alpha_{1}\left(1+Rd\right){\theta^{\prime }}^{2}+L\theta^{\prime }\phi^{\prime }+L\frac{\alpha_{2}}{\alpha_{1}}{\phi^{\prime }}^{2}}{\alpha_{1}\left(1+Rd\right){\theta^{\prime }}^{2}+Br\left(1+\frac{1}{\beta }\right){f^{\prime \prime }}^{2}+MBr\sin^{2}\alpha^{*}{f^{\prime }}^{2}+L\theta^{\prime }\phi^{\prime }+L\frac{\alpha_{2}}{\alpha_{1}}{\phi^{\prime }}^{2}}$$

where $N_{G}\left(=\frac{S_{G}T_{\infty }\nu }{k\Omega T_{0}}\right)$ indicates the entropy rate, $L\left(=\frac{RDC_{0}}{k}\right)$ the diffusion variables, and $\alpha_{2}\left(=\frac{C_{0}}{C_{\infty }}\right)$ the concentration ratio parameter.

## 5. Physical Quantities

### 5.1. Nusselt Number

The Nuselt number is defined as

(17) $$Nu_{x}=\frac{{rq_{w}|}_{z=0}}{k\left(T_{w}-T_{\infty }\right)},$$

with heat flux $q_{w}$ given by

(18) $$q_{w}=-\left(\frac{\partial T}{\partial z}\right)-\frac{16}{3}\frac{\sigma^{*}T_{\infty }^{3}}{k^{*}}\left(\frac{\partial T}{\partial z}\right).$$

From Equations (17) and (18) and dimensionless variables are obtained

(19) NuxRex−1/2=−(1+Rd)θ′(0).

### 5.2. Sherwood Number

We have

(20) $$Sh_{x}=\frac{{rj_{w}|}_{z=0}}{\left(C_{w}-C_{\infty }\right)},$$

with mass flux $j_{w}$ given by

(21) $$j_{w}=-D\left(\frac{\partial C}{\partial z}\right),$$

now we finally write

(22) ShxRex−1/2=−ϕ′(0).

Here, Rex(=ruwν), shows the local Reynold number.

## 6. Solution Methodology

The method NDSolve in “Numerical Differential Equations” allows us to get computational convergent solutions differential systems. The NDSolve technique deals with single differential systems and simultaneous differential systems. It also deals with partial differential systems. In the scheme of ordinary systems, there are unknown functions like Yi. These functions depend on a distinct “independent variable” x, which is the same for individual functions. It is a standard technique built in Mathematica 9.0 [37].

## 7. Validation of Result

Table 1 is prepared for the comparison of the present results with a limiting sense and past studies. Table 1 focuses on the assessment of the Nusselt number for different estimations of the Prandtl number (Pr) with studies [35,36]. The results are very good in agreement.

The above tabulated values are in reasonable agreement.

## 8. Discussion

Here, we applied the NDSolve method to develop computational outcomes for the given differential equations. Salient features of various parameters on velocity, concentration, Bejan number, entropy optimization, and temperature are studied. Velocity gradient and Nusselt and Sherwood numbers are numerically computed.

### 8.1. Velocity

**Figure 2:**
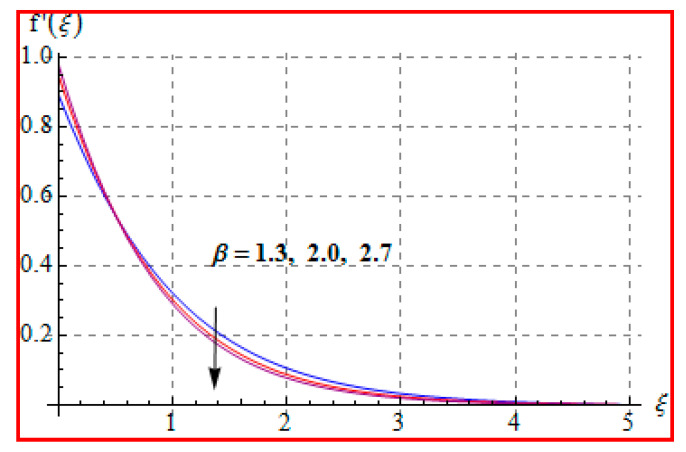
$f^{\prime }\left(\xi \right)$ against β.

**Figure 3:**
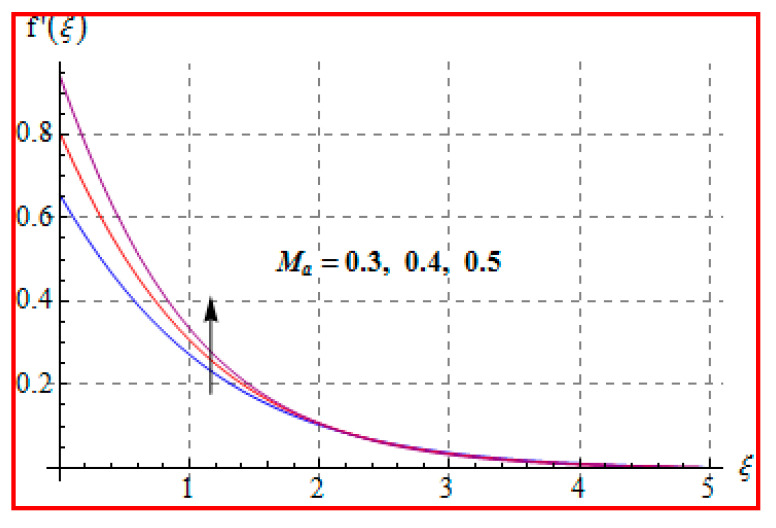
$f^{\prime }\left(\xi \right)$ against $M_{a}$.

**Figure 4:**
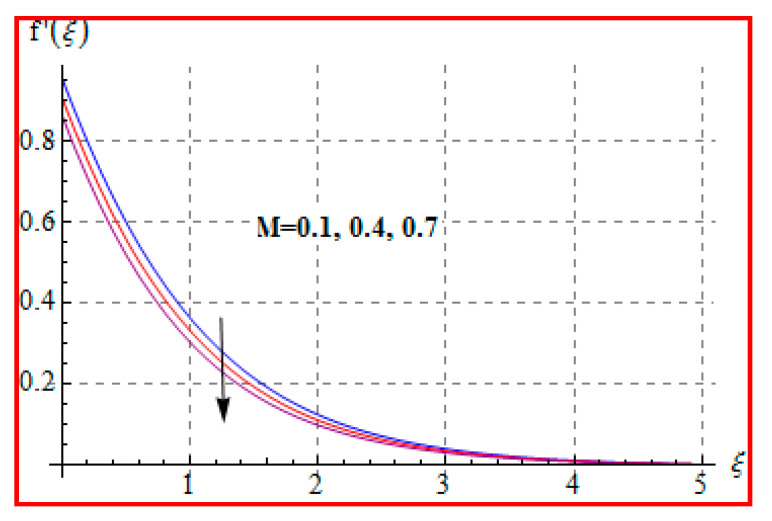
$f^{\prime }\left(\xi \right)$ against M.

**Figure 5:**
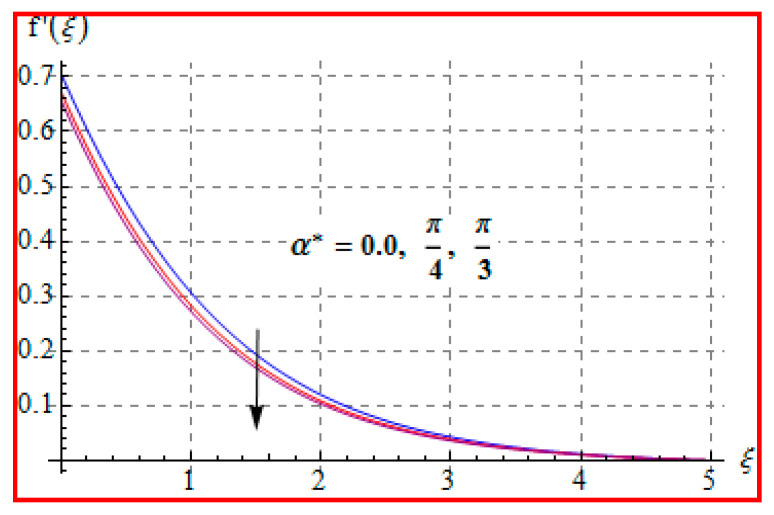
$f^{\prime }\left(\xi \right)$ against $\alpha^{*}$.

The influence of the Casson fluid parameter (β) on $f^{\prime }\left(\xi \right)$ is seen in Figure 2. Clearly, the velocity is decreased for higher (β). An increment in Casson fluid parameter yields more disturbances to fluid flow. Therefore, velocity is decreased. Physically, Casson fluid parameter (β) leads to resistance for fluid particles. Figure 3 is drawn to show the effect of ($M_{a}$) on $\left(f^{\prime }\left(\xi \right)\right)$. One can find that a higher ($M_{a}$) gives rise to velocity. Physically, a higher $M_{a}$ corresponds to less viscosity. A less viscous force has a tendency to speed up the fluid motion. Figure 4 reveals the outcome of (M) on $f^{\prime }\left(\xi \right)$. Here, velocity decreases against higher (M). In fact, the magnetic field applied in transverse direction opposes the transport phenomena. That is why the application of such a magnetic field yields a drag-like force, namely, the Lorentz force. This force acts in the opposite direction, which results in the reduction of velocity. Such an observation qualitatively agrees with the expectations. The influence of ($\alpha^{*}$) on $f^{\prime }\left(\xi \right)$ is portrayed in Figure 5. The results of $\alpha^{*}$ and M on f′ are similar in a qualitative sense.

### 8.2. Temperature

**Figure 6:**
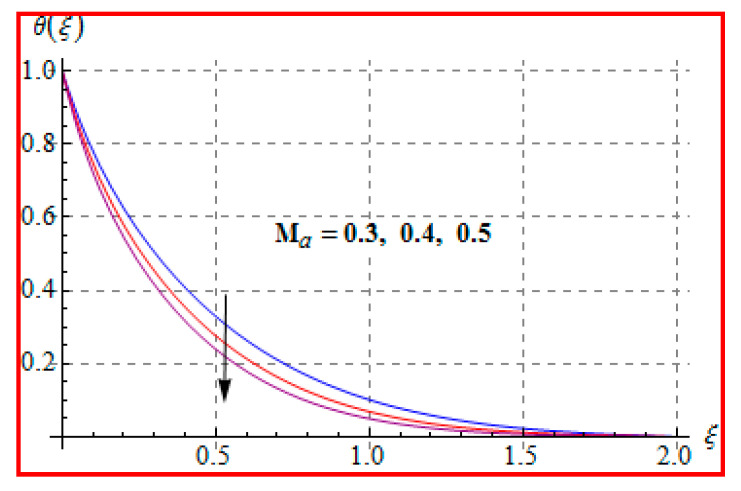
θ(ξ) against $M_{a}$.

**Figure 7:**
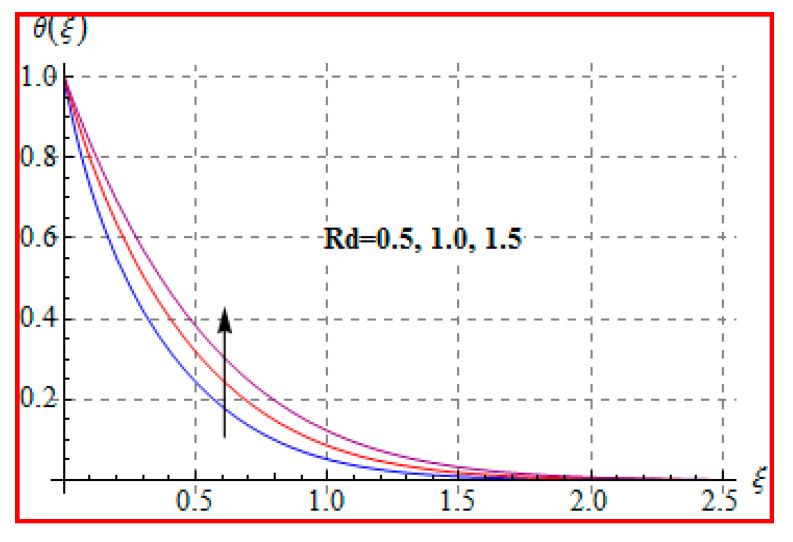
θ(ξ) against Rd.

**Figure 8:**
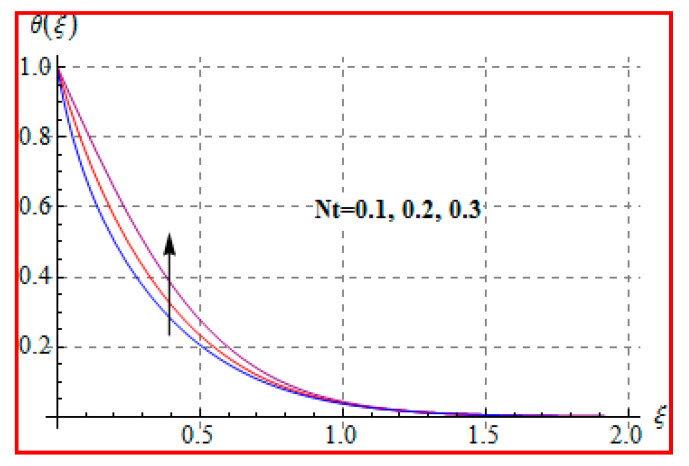
θ(ξ) against Nt.

**Figure 9:**
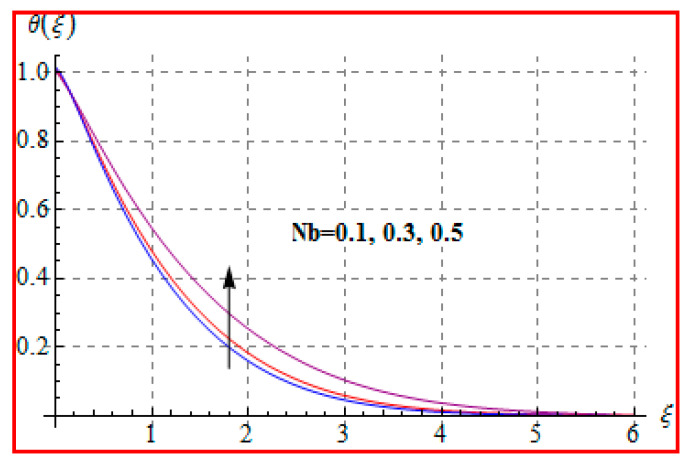
θ(ξ) against Nb.

**Figure 10:**
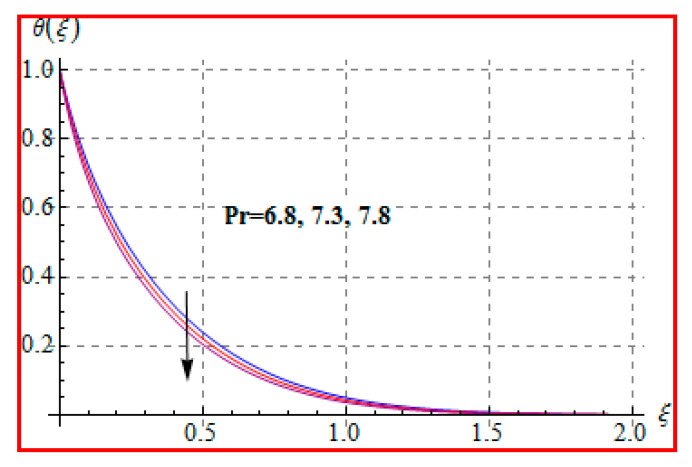
θ(ξ) against Pr.

**Figure 11:**
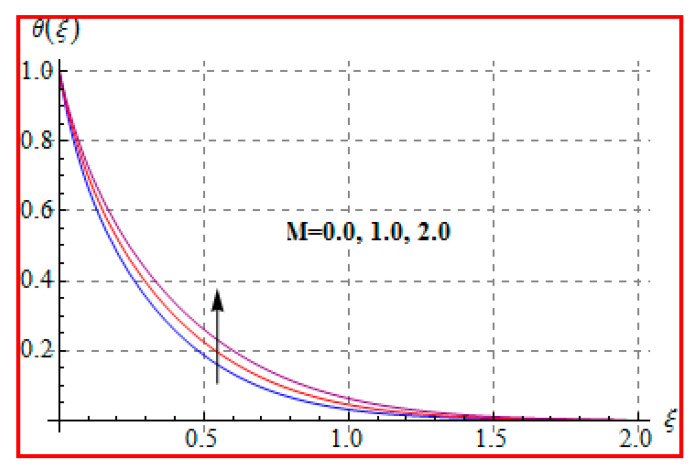
θ(ξ) against M.

**Figure 12:**
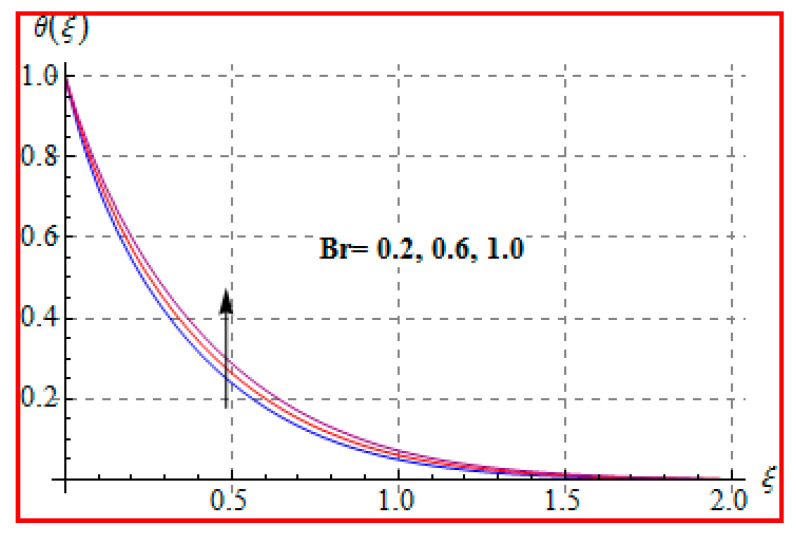
θ(ξ) against Br.

Figure 6, Figure 7, Figure 8, Figure 9, Figure 10, Figure 11 and Figure 12 are sketched for characteristics of ($M_{a}$), (Rd), (Nt), (Nb), (Pr), (M), and (Br) on temperature (θ(ξ)). Figure 6 examines the effect of ($M_{a}$) on θ(ξ). The temperature is increased for a higher estimation of ($M_{a}$). There is no doubt that the velocity grows in view of less viscosity for a higher ($M_{a}$). Temperature is the average kinetic energy of a fluid particle. As a result, the temperature enhances for a higher $M_{a}.$ The behavior of thermal radiation parameter (Rd) on (θ(ξ)) is portrayed in Figure 7. For higher radiation parameter, the temperature θ(ξ) increases. Physically, the rise of Rd has the tendency to enhance conduction impact and to increase temperature at each point away from the surface. In other words, there is a higher heat flux for a larger Rd. Figure 8 and Figure 9 displayed the behaviors of (Nt) and (Nb) on θ(ξ). It is clear from Figure 8 and Figure 9 that the temperature augmented for higher (Nt) and (Nb). Clearly, the velocity grows at a higher $N_{t\;}$ and $N_{b}$. These parameters lead to an enhancement of thermal-boundary layer thickness. In view of such reasons, the temperature is enhanced. The behavior of (Pr) on temperature is shown in Figure 10. For higher (Pr), thermal diffusivity reduces and consequently θ(ξ) decreases. As expected, this is in accordance with the fact that when θ is at higher $P_{r}$, the fluid has a thinner thermal boundary layer and it yields more temperature gradient. The velocity reduces and momentum boundary layer thickness. The impact of (M) on temperature is illustrated in Figure 11. Clearly, the fluid temperature accelerates by increasing the strength of the magnetic field. This leads to the argument that an applied magnetic field tends to heat the liquid, and thus heat transfer from the disk enhances. All of this occurs due to Lorentz force in view of the transverse applied magnetic field opposing the transport phenomena and slowing down the fluid motion. No doubt, the magnetic can be employed as a useful agent for controlling the flow and heat transfer characteristics. Figure 12 displays the influence of Brinkman number on temperature (θ(ξ)). One can find that the temperature increased against (Br). An augmentation in Br creates more kinetic energy that enhances the temperature field. Physically, Eckert number in Brinkman number is responsible for the increase in temperature. Such an increase in temperature through higher Eckert number is due to more pronounced viscous heating. Obviously, a high Eckert number gives rise to wall cooling and thus transfer of heat to fluid, so fluid temperature rises.

### 8.3. Concentration

**Figure 13:**
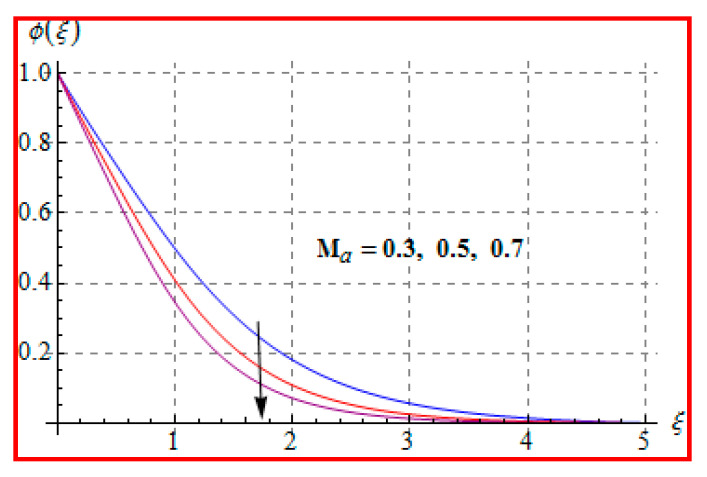
ϕ(ξ) against $M_{a}$.

**Figure 14:**
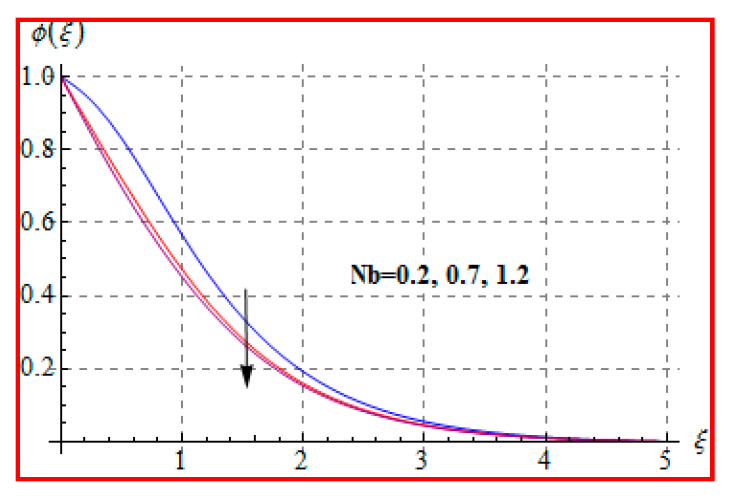
ϕ(ξ) via Nb.

**Figure 15:**
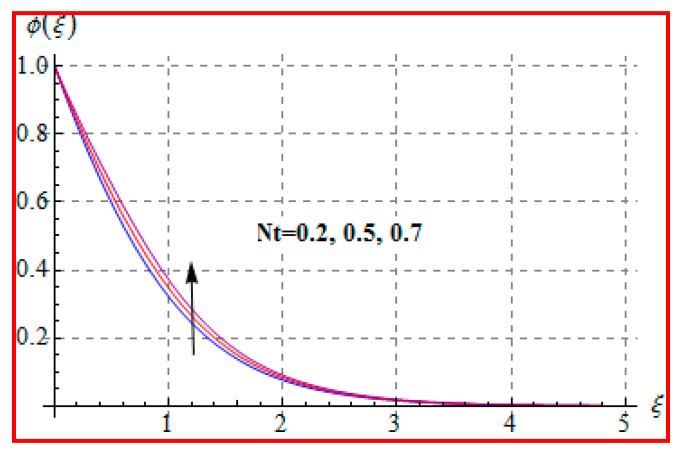
ϕ(ξ) against Nt.

**Figure 16:**
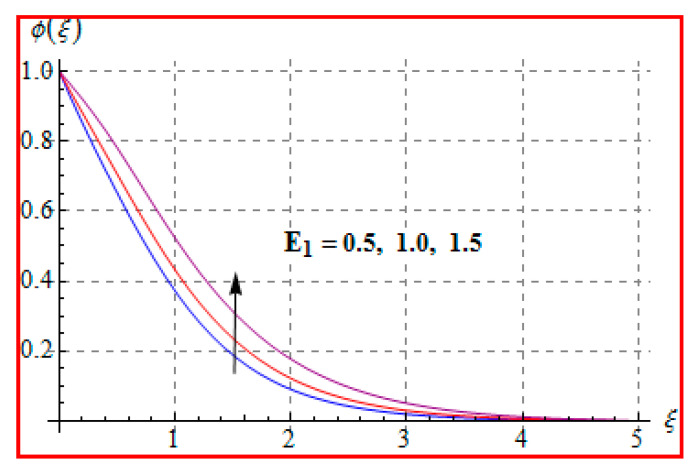
ϕ(ξ) via $E_{1}$.

**Figure 17:**
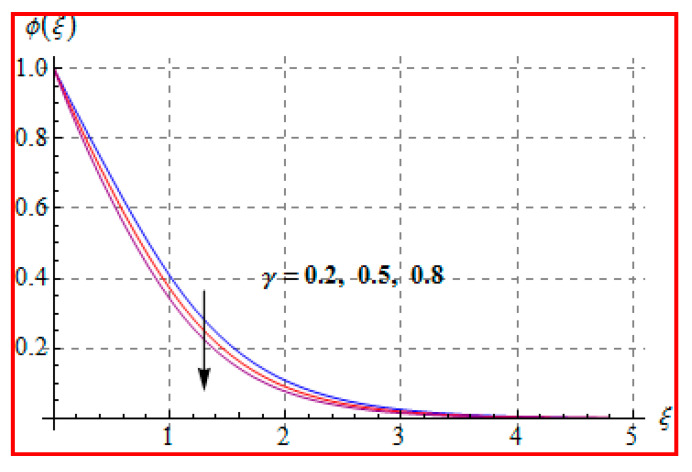
ϕ(ξ) against γ.

**Figure 18:**
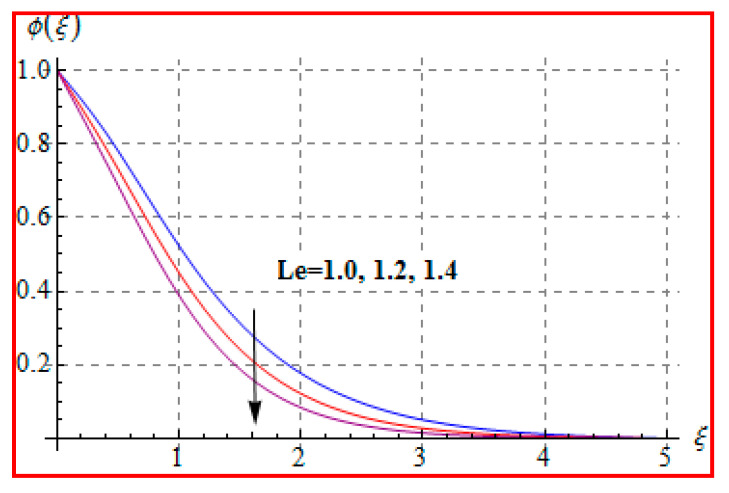
ϕ(ξ) against Le.

Characteristics of Marangoni number $\left(M_{a}\right)$ on ϕ(ξ) are shown in Figure 13. For higher $\left(M_{a}\right)$, the concentration decays because of surface tension produced by temperature and concentration gradients. The reduction in concentration is accompanied by simultaneous decay in the concentration of boundary layer thickness. Figure 14 and Figure 15 display the effects of (Nb) and (Nt) on ϕ(ξ). Opposite impacts of (Nb) and (Nt) are seen on concentration. Such behavior totally depends upon the nanoparticles volume fraction. When Brownian motion parameter $N_{b}$ enhances, nanoparticles volume fraction decays and so ϕ reduces. On the other hand, for a higher $N_{t}$, the volume fraction of nanoparticles increases. Figure 16 exhibits the effect of ($E_{1}$) on ϕ(ξ). In fact, higher activation energy parameter reduces the modified Arrhenius function, which consequently increases the generative chemical reaction. As a result, the concentration is augmented. Figure 17 shows the effect of (γ) on concentration. Clearly, for a higher (γ) the liquid becomes thicker, and therefore concentration decreases. This happens in view of higher viscosity. Figure 18 is provided to study the effect of (Le) on concentration. One can find that concentration decays versus (Le). It is noticed that high Lewis number ($L_{e}$) decays the nanoparticles volume fraction. Lewis number is the ratio of thermal diffusivity to mass diffusivity. A higher value of Lewis number enhances the thickness of the thermal boundary layer. The concentration boundary layer thickness decays.

### 8.4. Entropy Optimization and Bejan Number

**Figure 19:**
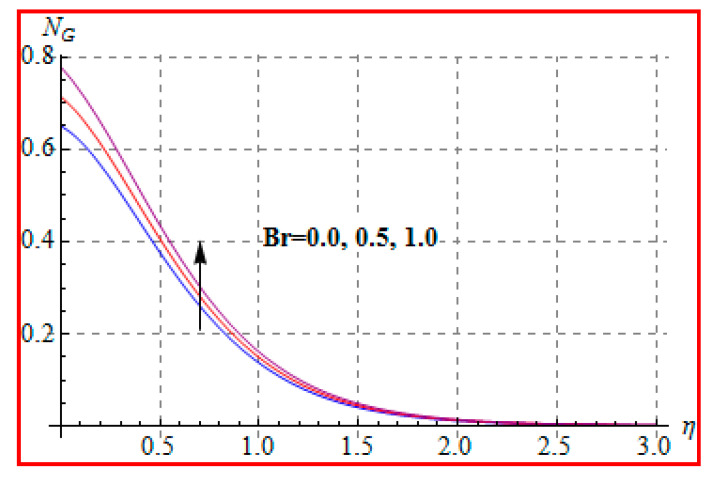
$N_{G}$ against Br.

**Figure 20:**
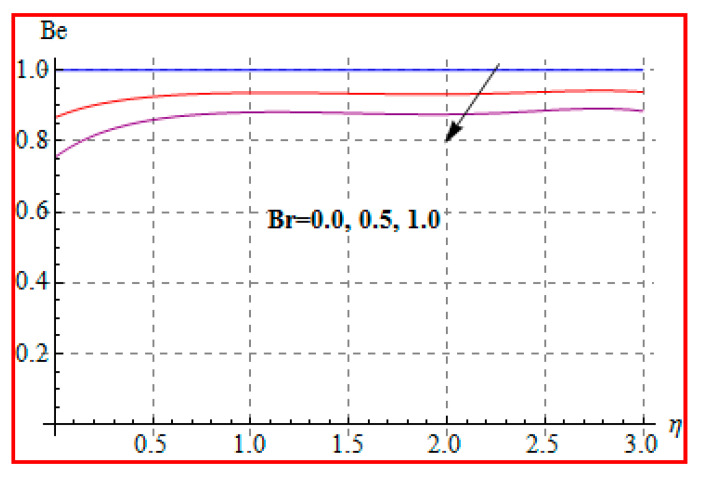
Be via Br.

**Figure 21:**
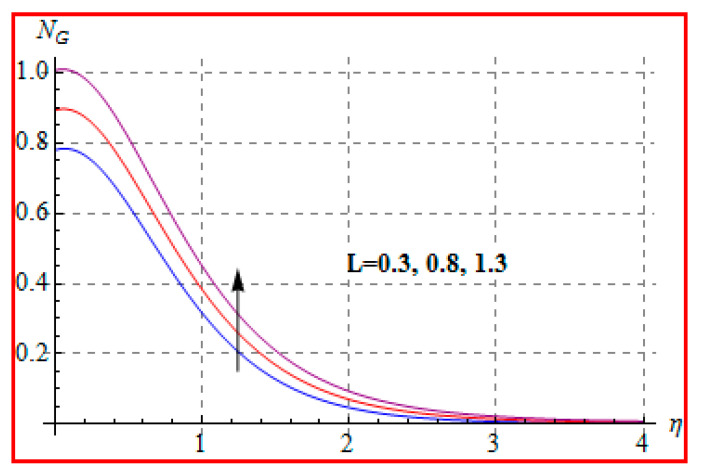
$N_{G}$ via L.

**Figure 22:**
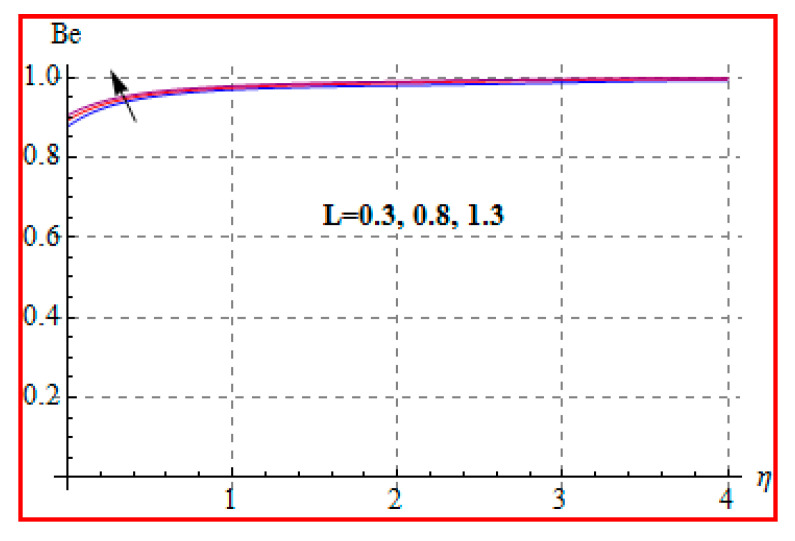
Be via L.

**Figure 23:**
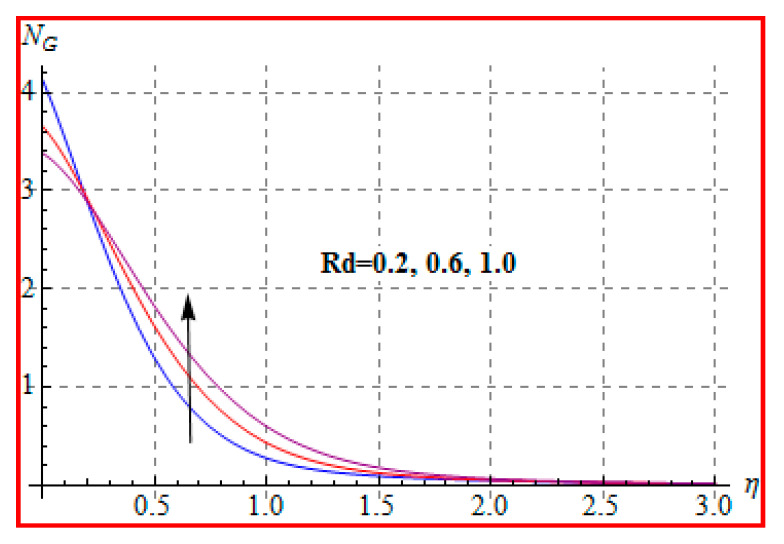
$N_{G}$ via Rd.

**Figure 24:**
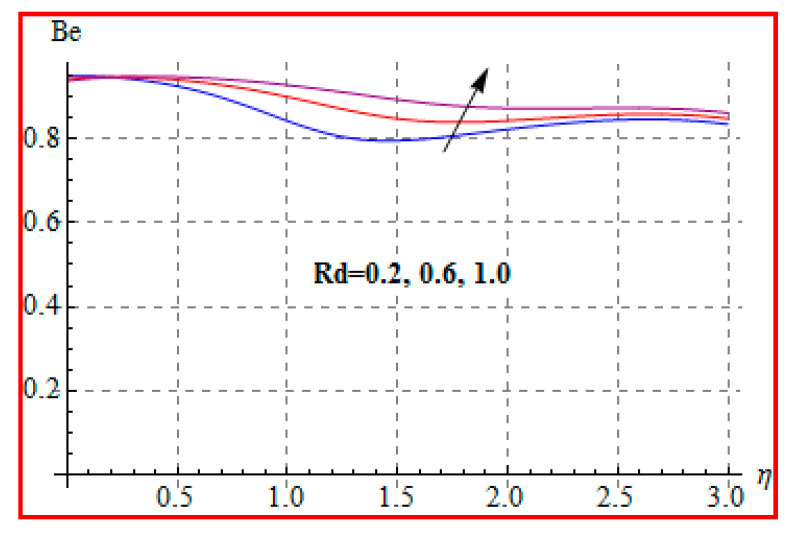
Be via Rd.

**Figure 25:**
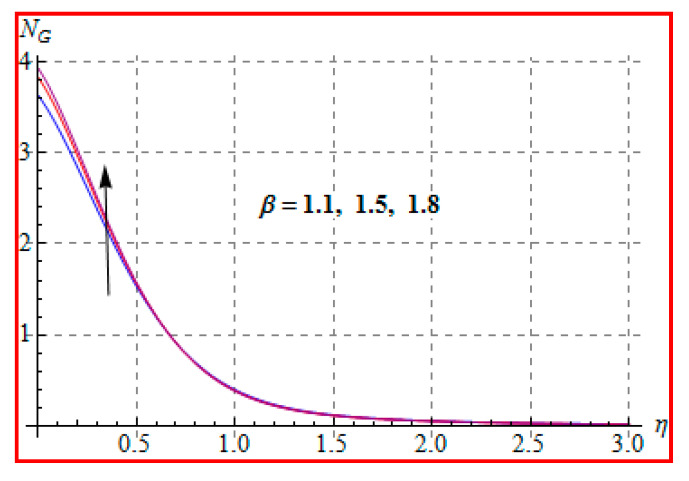
$N_{G}$ via β.

**Figure 26:**
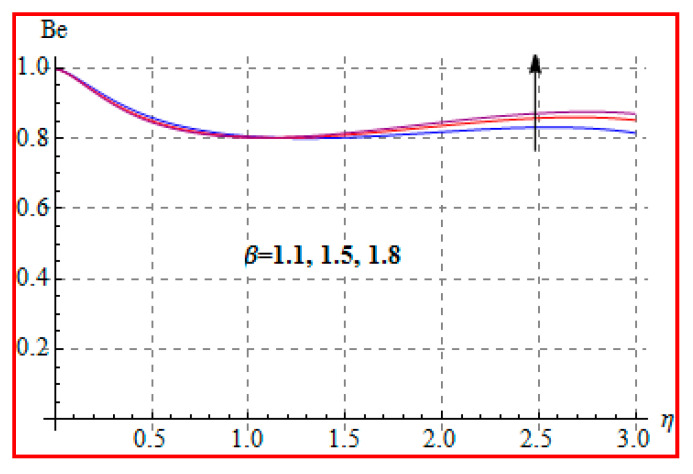
Be via β.

Characteristics of Brinkman (Br) number on $N_{G}$ and Be are elucidated in Figure 19 and Figure 20. In fact, Eckert number in definition of Br increases the temperature, and consequently entropy enhances. Bejan number Be has the opposite scenario for higher Br. In other words, there is a direct relationship of Br with heat transfer by molecular diffusion and heat generated by fluid friction. For higher Br, there is more production of heat in the system. This raises the system disorderliness. Figure 21 and Figure 22 elucidate the characteristics of (L) on $N_{G}$ and Be. Clearly, both $N_{G}$ and Be are increased for a higher diffusion parameter (L). Here, more disorderliness of the system is due to a higher diffusivity in the fluid particle. This fact leads to an increase in the diffusive variable, the entropy generation, and the Bejan number. Figure 23 and Figure 24 are displayed to study the variation of (Rd) on $N_{G}$ and Be. For a higher estimation of (Rd), the $N_{G}$ increases. In fact, for a higher (Rd) the radiation enhances. As a result, there is a growth in temperature, and therefore the entropy rate increases. Be also boosts up via higher (Rd), because of the fact that heat transfer irreversibility dominates over total entropy optimization. Influence of (β) on $N_{G}$ and Be is explained in Figure 25 and Figure 26. Clearly, for a higher approximation of (β), both $N_{G}$ and Be are augmented. Actually, for a higher estimation of (β), the more resistive force is created in flow region, which improves the thermal energy due to the collision of liquid particles. As a result, the entropy generation is enhanced.

### 8.5. Engineering Quantities

Salient features of different variables on Nusselt and Sherwood numbers are discussed in Table 1 and Table 2.

#### 8.5.1. Nusselt Number

Table 2 elucidates the numerical approximation of $\left(Nu_{x}\right)$ for $\left(M_{a}\right)$, (Nb), (Nt), and (Pr). Nusselt number increases via (Nb), while the reverse is observed through (Nt). For a higher $\left(M_{a}\right)$, the Nusselt number is decreased. $\left(Nu_{x}\right)$ increases against higher (Pr)**.** There is a thinner thermal boundary layer for higher $P_{r}$. As a result, the rate of heat diffusion enhances.

#### 8.5.2. Sherwood Number

Salient features of different variables like (Nt), (Nb), and (Le) on Sherwood number ($Sh_{x}$) are shown in Table 3. Here, ($Sh_{x}$) boosts up for higher estimations of (Nb) and (Le). Sherwood number ($Sh_{x}$) is a decreasing function of (Nt).

## 9. Conclusions

The main points of the presented analysis are listed below.

- There is a reduction in velocity for a higher Casson fluid parameter and magnetic field. However, the scenario is different for velocity through the Marangoni number.
- Temperature is enhanced for both thermophoresis and Brownian motion parameters.
- An increasing trend of temperature is noticed for a higher Marangoni number, radiation and magnetic field, and Brinkman number.
- Prandtl number reduces the temperature.
- Concentration for Lewis and Marangoni numbers is opposite to that of the activation energy variable.
- Reverse behavior of Brownian motion and thermophoresis for concentration is noted.
- Bejan number for radiation and Brinkman number have opposite outcomes.
- A qualitative, similar effect of entropy generation and Bejan number is noted with respect to the diffusion parameter.
- Sherwood number for Brownian motion and Lewis number is opposite when compared with the thermophoresis parameter.

The present attempt is basic, and modeling and simulation about such problems can be extended for the Williamson fluid subject to Soret and Dufour effects, viscous dissipation with rheological impact, and porous medium employing modified Darcy’s law. Nothing is yet known about such facts in the existing literature.

## Figures and Tables

**Table 1:** Comparison of Nusselt number with [35,36].

PR.	SITHOLE ET AL. [35]	OLANREWAJU ET AL. [36]	PRESENT RESULT
0.5	0.21441547	0.214368	0.214363
0.7	0.24976956	0.250142	0.250139
1.0	0.28782508	0.289161	0.289142
2.0	0.35519994	0.356176	0.356145

**Table 2:** Variation of Physical Parameters against $M_{a}$.

$M_{a}$	Nb	Nt	Pr	$Nu_{x}$
0.0	0.5	0.4	0.5	1.00091
0.2				0.983521
0.4				0.974563
0.3	0.1	0.4	0.5	0.895316
	0.6			0.934756
	1.0			0.979105
0.3	0.5	0.2	0.5	0.99962
		0.5		0.998332
		0.8		0.997925
0.3	0.5	0.4	0.5	0.978231
			1.0	0.989432
			1.5	0.994214

**Table 3:** Variation of Physical Parameters against $Sh_{x}$.

Nb	Nt	Le	$Sh_{x}$
0.3	0.2	0.5	0.503491
0.6			0.615362
0.9			0.676212
0.2	0.2	0.5	0.514252
	0.5		0.374541
	0.8		0.234561
0.2	0.2	0.5	0.534567
		1.0	0.697349
		1.5	0.816734
